# A Piezoresistive Sensor to Measure Muscle Contraction and Mechanomyography

**DOI:** 10.3390/s18082553

**Published:** 2018-08-04

**Authors:** Daniele Esposito, Emilio Andreozzi, Antonio Fratini, Gaetano D Gargiulo, Sergio Savino, Vincenzo Niola, Paolo Bifulco

**Affiliations:** 1 Department of Electrical Engineering and Information Technologies, University “Federico II” of Naples, Via Claudio, 21-80125 Napoli, Italy; daniele.esposito@unina.it (D.E.); emilio.andreozzi@unina.it (E.A.); 2 Istituti Clinici Scientifici Maugeri s.p.a.—Società benefit, Via S. Maugeri, 4-27100 Pavia, Italy; 3 School of Life and Health Sciences, Aston University, Birmingham B4 7ET, UK; a.fratini@aston.ac.uk; 4 The MARCS Institute, Western Sydney University, Penrith, NSW 2751, Australia; g.gargiulo@uws.edu.au; 5 Department of Industrial Engineering, University “Federico II” of Naples, Via Claudio, 21-80125 Napoli, Italy; sergio.savino@unina.it (S.S.); vincenzo.niola@unina.it (V.N.)

**Keywords:** force sensitive resistor, muscle contraction, electromyography, mechanomyography, prosthesis control, human machine interface

## Abstract

Measurement of muscle contraction is mainly achieved through electromyography (EMG) and is an area of interest for many biomedical applications, including prosthesis control and human machine interface. However, EMG has some drawbacks, and there are also alternative methods for measuring muscle activity, such as by monitoring the mechanical variations that occur during contraction. In this study, a new, simple, non-invasive sensor based on a force-sensitive resistor (FSR) which is able to measure muscle contraction is presented. The sensor, applied on the skin through a rigid dome, senses the mechanical force exerted by the underlying contracting muscles. Although FSR creep causes output drift, it was found that appropriate FSR conditioning reduces the drift by fixing the voltage across the FSR and provides voltage output proportional to force. In addition to the larger contraction signal, the sensor was able to detect the mechanomyogram (MMG), i.e., the little vibrations which occur during muscle contraction. The frequency response of the FSR sensor was found to be large enough to correctly measure the MMG. Simultaneous recordings from flexor carpi ulnaris showed a high correlation (Pearson’s r > 0.9) between the FSR output and the EMG linear envelope. Preliminary validation tests on healthy subjects showed the ability of the FSR sensor, used instead of the EMG, to proportionally control a hand prosthesis, achieving comparable performances.

## 1. Introduction

Measurement of muscle contraction is of interest to many medical branches (e.g., neurology, orthopedics, rehabilitation, sport medicine, etc.) [1]. In particular, dynamic measurement of voluntary muscle activity is widely used for active prosthesis control and, more generally, for man-machine interface. For these applications, sensors should be non-invasive, proportional to contraction level, effective, easy to wear, long-lasting, etc. [2,3].

In general, muscle activity can be monitored by recording either the related electrical or mechanical phenomena. Surface electromyography (EMG) is, by far, the main non-invasive technique used to assess muscle functioning [1]. EMG is based on recording the electric potential connected to muscular fibers’ depolarization, which is the trigger signal for fibers’ shortening and muscle contraction. Electrodes and biopotential amplifiers are needed to record either surface or intramuscular EMG. Surface EMG signals are widely used as a control signal for active prosthetic devices [2]. Surface EMG requires stable electrode placement and good electrical contact with skin. Conductive gel facilitates and stabilizes the skin/electrode electrical interface, but for long-lasting application, dry electrodes should be used [4]. Raw EMG signals are usually pre-processed (e.g., rectified and low-pass filtered) to extract its envelope, which is roughly proportional to the muscle contraction level. The quality of surface EMG also depends on properties of the skin (e.g., dryness, cleanness, visceral fat, etc.), the relative motion between electrodes and muscle fibers (motion artifact), and crosstalk with other biopotentials (e.g., adjacent muscles’ activations). Recordings are very sensitive to external electromagnetic interference and also to other sources of noise, such as the electrochemical reactions at the electrode interface, the electronic noises which arise from an electrode-amplifier impedance mismatch, etc. [1,5,6].

Different mechanical features can also be used to monitor muscle activation. Muscle contraction is generally associated with muscle shortening, increase of cross-section areas, increase of stiffness and tension, mechanical vibrations, and other mechanical parameters. Mechanomyography (MMG) can be regarded as the mechanical counterpart of the EMG [7,8], and is based on the recording of muscular vibrations produced by an active muscle. It can be used as a monitor of muscle stiffness and can be related to muscle force exertion [9]. The amplitude of the MMG signal may be related to the number of active motor units (i.e., motor unit recruitment) [8]. Different types of sensors can be used to measure the MMG signal: condenser microphones [8,10,11], piezoelectric contact sensors, accelerometers [8,12,13,14,15], and, more recently, laser distance sensors [8], placed in contact with the patient’s skin. Tensiomyography is another technique used to estimate a muscle’s mechanical properties, and it does so by measuring the radial enlargement of the muscle belly and detects muscle contractile properties [16,17,18].

Mechanical muscle contraction can be measured in many ways [3], including:

- Strain gauges [19]: muscle contractions which cause direct stretching of the sensor;
- Change of electrical impedance of the muscles [20]: changes to global muscle resistivity when it goes from a resting state to an activity state, due to blood afflux in the muscles;
- Muscle circumference sensor [21,22]: where muscular contraction is proportional to the changing of cross-sectional areas of the muscles, around which the sensor is positioned;
- A resonance-based active-muscle stiffness sensor [23]: where piezoelectric probes are used to measure stiffness changes in muscles;
- Ultrasound scanners [9,24]: where ultrasound probes are employed to evaluate the morphological changes in muscle thickness or displacement;
- A small permanent magnet fixed on the skin, in conjunction with a Hall effect device, used to measure changes in muscle dimension [25];
- Pneumatic sensors [26]: muscular activity detected by measuring changes in air pressure in an air-bladder contacting the muscle;
- Change in optical properties [27]: LEDs and photodiodes can be combined to detect muscle contraction by measuring the backscattered light from the muscle tissue;
- Textile pressure sensors enclosed in garments [28].

In particular, some previous studies [29,30] reported the use of force-sensitive resistors (FSR) to acquire information about muscle activity, however, they could only provide qualitative results or simple information about on-off muscle activation. They used bare FSR sensors laid onto the patient’s skin, and no quantitative comparisons with other, more well-assessed signals proportional to the intensity of muscle contractions (as the EMG) were made. The use of a very simple voltage divider conditioning circuit produced intense drift and differences in sensitivity over the operative range, as demonstrated by [31].

It is worth emphasizing that EMG reflects the electrical, not mechanical events of muscle contraction. In fact, EMG represents the propagation of motor unit action potentials along muscle fibers, which is only the initiator of the muscle mechanical activity, followed by chemical and mechanical events [32] which take place before the muscle actually exerts force. The time lag between the EMG onset and the beginning of force generation is defined as the electromechanical delay (EMD) [32]. Similarly, latency between the end of the EMG signal and the beginning of force fading occurs during muscle relaxation [33], and it is known as the relaxation electromechanical delay (r-EMD) [34]. Again, chemical and mechanical events occur [32]. Moreover, while muscle contraction is an active process, relaxation is passive, and other biomechanical events (e.g., action of antagonist muscle and/or other external counteracting forces) have to be considered also. Simultaneous recordings of EMG, MMG, and force (or torque) can accurately evaluate such electromechanical delays [35,36,37].

In the vast majority of applications involving measurement of muscular contraction levels (such as prosthesis control), EMG-concise information, such as the EMG linear envelope (EMG-LE) is still used. However, the aforementioned problems related to EMG collection (i.e., electrodes, interferences, motion artifacts, etc.) have stimulated interest in other types of sensors, such as those sensitive to the muscle mechanical activity, but nothing has proved so effective to offer a reliable replacement for the EMG-LE. Factors such as ease of use, stable positioning on patient, size, robustness, durability, wearability, cost, energy transmitted to the patient, electrical connections with the patient, etc. have hindered its extensive usage. In exchange, a new muscle contraction sensor that overcomes many of these limitations is presented. It is based on a slim force-sensitive resistor mounted on a purposefully designed mechanical coupling system, driven by a specific conditioning circuit. The new sensor is extremely simple and easy to use, and is able to record both muscle cross-sectional changes and muscle oscillations, thus providing signals comparable to the EMG-LE and the MMG. The new sensor went through a preliminary test as a replacement of an EMG sensor to control an active hand prosthesis.

## 2. Materials and Methods

### 2.1. Sensor Design

**Figure 1:**
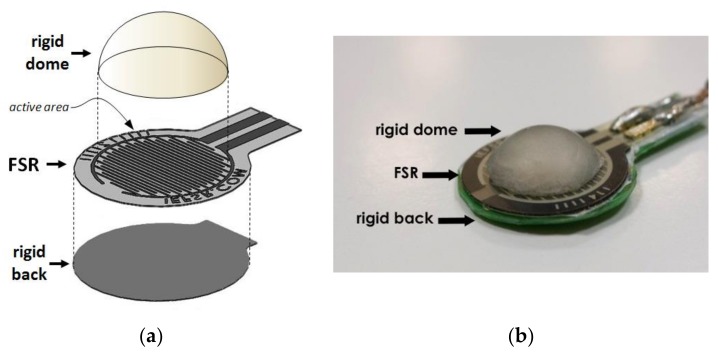
(**a**) Exploded view of the mechanical components of the force-sensitive resistor (FSR)-based muscle sensor. (**b**) A picture illustrating the FSR sensor and the rigid dome mounted above.

Muscle contraction is associated with volumetric and stiffness changes, which exert radial forces (or pressures). A force-sensitive resistor (FSR) placed on a patient’s skin in correspondence with a muscle belly was used to sense contraction. Generally, FSRs consist of a conductive polymer, which changes its resistance when a force is applied to its surface. They can be made small and very thin (e.g., less than 0.5 mm), offer good shock resistance, can operate in moderately hostile environments, and are low-cost. However, there should only be concentrated and uniformly distributed force within the FSR active (or sensing) area for reliable use of the FSR. The assembling of the Interlink FSR [38] includes perimetral spacers that separate the two membranes holding the metallic contacts and the conductive polymer. A direct application of the FSR on skin to sense muscle contraction proved to be quite unsatisfactory. The mere sensor, without any mechanical coupler, provided uncertain and unreliable results. Contact with the patient’s skin was unstable and uncertain—the perimetral spacers of the FSR sensor transmits part of the applied force directly to the back of the sensor without involving the sensing area, and prevents the membrane with electrical contacts from properly flexing onto the resistive polymer layer. A specific mechanical coupler was designed in response to these drawbacks (see Figure 1). A rigid spherical cap, made of acrylic resin, provides advantageous force transmission to FSR. The spherical cap base was glued onto the FSR’s sensitive area (leaving out the perimetral spacers) and its convex part was made to face the patient’s skin. When the sensor is applied onto the patient, the dome creates a little subsidence that gently but firmly attaches to the skin. Furthermore, a flat, rigid sheet of plastic was attached to the back of the sensor to prevent improper bending. The elastic modulus of both the spherical cap and the back support were much higher than that of skin and muscle. The mechanical coupler provides a much more convenient and reliable muscle force transmission to FSR.

The assembly of the FSR and the mechanical coupler can be held in place onto a patient’s skin by a belt or other fastening methods (e.g., scotch tape). The increase of muscular transverse section during contraction, as well as the resultant skin stretching, impresses uniform pressure on the FSR active area via the rigid spherical cup. Furthermore, the small mechanical vibrations generated during muscle contraction (i.e., the mechanomyography MMG signal) are suitably transmitted to the FSR sensor.

### 2.2. Sensor Conditioning

The use of conductive polymer composites as force sensors in robotic and biomedical applications [39,40] has been limited due to their low accuracy and repeatability in measuring absolute force (or pressure) compared to load cells. Some recent studies have modelled in detail the rheological behaviour of the insulating polymer matrix in which conductive particles are dispersed [31,41], and highlighted the role of the voltage across the sensor. When an FSR is subjected to constant loading for an extended period of time, mechanical creep behaviour can be observed in the physical dimensions of the specimen due to the rheological characteristics of the polymer. The creep affects the inter-particle separation, as well as the electrical resistance of FSR (see quantum tunneling operation mode [42])—this produces a drift of the sensor output. However, the sourcing voltage across the FSR sensor plays an important role in the output drift.

**Figure 2:**
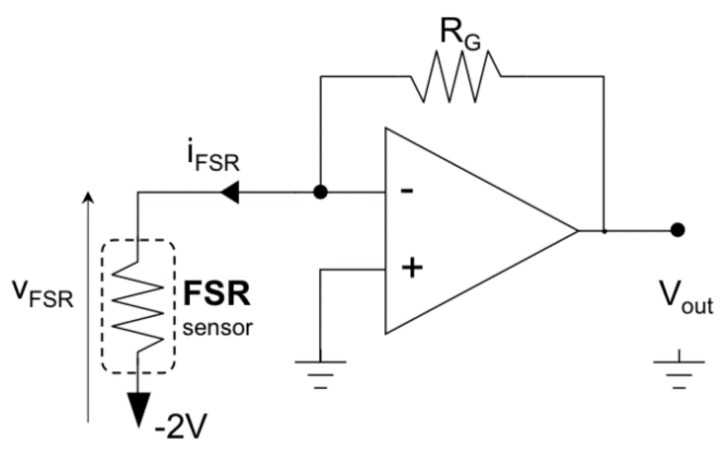
Conditioning circuit for the FSR muscle sensor.

The sensor datasheet [38] primarily reports a simple voltage divider circuit (a series of FSR and fixed resistors) for FSR conditioning. The voltage divider configuration [29] is mostly used for its simplicity, and is often supplied by the voltage available from microcontroller boards (e.g., 5 V). However, the voltage divider configuration does not ensure a constant voltage across the sensor (it may swing from a few millivolts up to 5 V)—such configurations increase and complicate sensor drift, yields sensitivity degradation, and provides much poorer measurement accuracy and repeatability. Thus, using voltage divider conditioning circuits have been discouraged [31], and a constant voltage should be used instead. The FSR conditioning circuit was designed using an op-amp trans-impedance amplifier (see Figure 2). This circuit maintains a constant voltage across the FSR, performs a current-to-voltage conversion, and makes the FSR sensitivity constant over a wide input force range. It was observed that if the FSR voltage is about a hundred mV (140 mV for the Interlink FSR 402 sensor [41]) there is a non-linear relationship between the FSR current and voltage. Furthermore, it was observed that by increasing the voltage, the drift would tend to reduce [31]. However, joule self-heating of the sensor suggested reduction of the supply voltage, and the non-linear phenomena of sensitivity degradation [39] could also be observed when an FSR, supplied with a voltage greater than 2 V [31], was subjected to cyclic loading. Therefore, a good compromise to minimize drift and preserve sensitivity is to supply the FSR sensor with a constant voltage of 2 V.

With reference to muscle contraction sensing, the large time constant of the mechanical creep and the sensor drift (about 500 s [31]) should be compared to muscles’ activation intervals, which are usually much shorter. However, it is important for the sensor to have stable sensitivity over time [31,39] so that repeated or cyclic muscle contractions can be monitored. In conclusion, FSR sensor drift and sensitivity degradation, although not eliminated, can be effectively limited by means of appropriate FSR conditioning circuits, as adopted in this study.

### 2.3. FSR Static and Dynamic Test

Static calibration of the FSR sensor was performed to measure the relationship between force and voltage output (using the conditioning circuit of Figure 2). Different weights were applied onto the FSR sensor, and the resulting voltages were recorded. The weights were sequentially and perpendicularly applied to the active area of the sensor (on the top of the spherical cup), while the sensor was placed on a precision electronic scale that measured the actual force impressed. In addition, FSR sensor outputs were recorded for 200 s after the application of static loads in order to measure the actual drift achieved by the conditioning circuit. Drift was expressed as a percentage of variation from the expected value, according to Equation (1):

(1) $$Drift\left(t\right)=\frac{V_{FSR}\left(t\right)-V_{FSR}\left(0\right)}{V_{FSR}\left(0\right)}\times 100$$

where *Drift*(*t*) is the normalized voltage drift in percentages; *V_FSR_*(*t*) is the voltage output of the FSR sensor at time *t*, and *V_FSR_*(*0*) is the sensor voltage immediately after the application of stress (i.e., the expected value).

**Figure 3:**
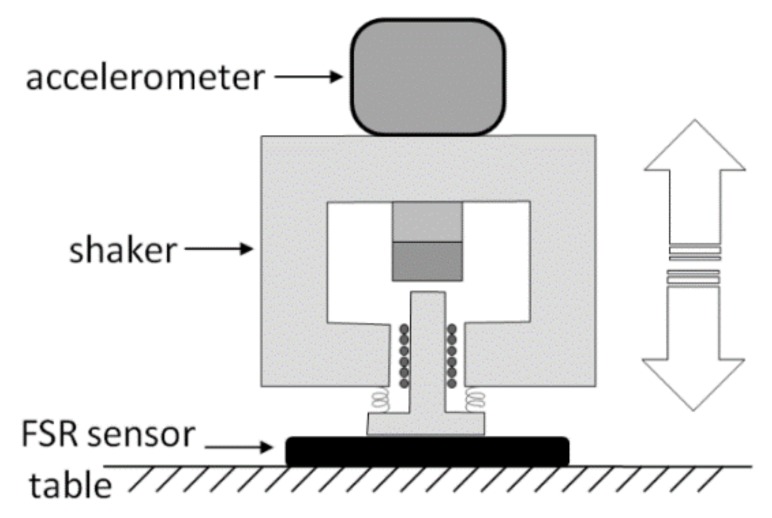
Experimental set-up to measure the frequency response of the FSR sensor.

The frequency response of the sensor was experimentally evaluated in order to test its ability to record rapidly varying signals, such as the MMG. A specific measurement set-up (see Figure 3) was designed and realized to practically measure the FSR sensor amplitude and phase response at different mechanical frequencies. A little electrodynamic shaker, supplied by a signal generator (Hewlett Packard HP 33120A) was mounted on top of the sensor, placed on a table. A precision accelerometer (PCB Piezotronics ICP 352B, sensor signal conditioner model 480C02) was fixed by screws onto the shaker (at the center of the upper part) to measure the acceleration of its mass. The weight of the shaker and accelerometer assembly was 315 grams. The actual force applied onto the sensor was obtained by multiplying the mass by the acceleration.

The shaker was driven by a sinusoidal voltage of increasing frequency (within the range of 1–2000 Hz, using 50 Hz steps) while the accelerometer signal and the voltage output of the sensor were sampled by means of an acquisition board (National Instruments NI USB-4431). Both signals were acquired at 100 kHz sampling frequency with 24-bit precision. The modulus of the FSR frequency response was determined by computing the ratio between the sensor output and the actual applied force, computed from the accelerometer data. The phase of the FSR frequency response was determined by measuring the time lag between the positive-slope zero-crossings of the applied sinusoidal force and of the FSR voltage output.

### 2.4. Comparison with the EMG Signal and Hand Prosthesis Control

**Figure 4:**
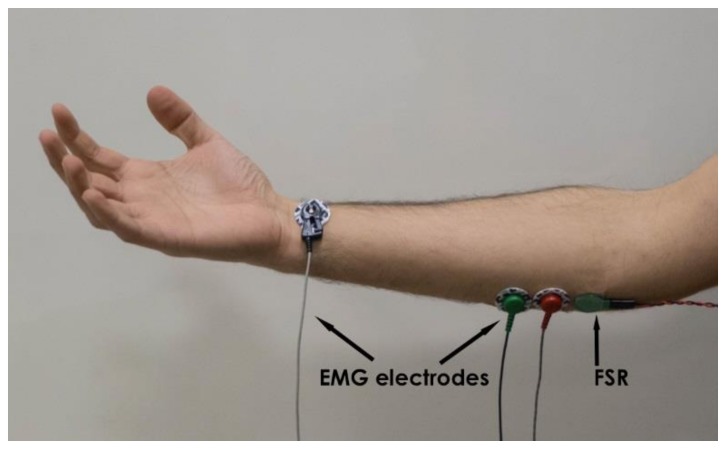
Electromyography (EMG) electrodes and FSR sensor placed on patient’s muscle.

Simultaneous measurements of EMG and the FSR sensor output were carried out on the forearm muscle of five healthy volunteer subjects (males aged 25–50) in order to achieve a quantitative comparison between the EMG-LE and the FSR sensor. As reported in the introduction, the muscular electro-mechanical delay needed to be taken into account when comparing the two signals. Tests were performed on the flexor carpi ulnaris, a muscle involved in prosthetic hand control [2,3]. EMG electrodes and the FSR sensor were placed closed to each other on the belly of the muscle, as shown in Figure 4. The EMG signal was acquired by means of a biopotential amplifier (Biomedica Mangoni BM623) enabling a hardware 10–500 Hz band-pass filter. The two signals were simultaneously acquired at 10 kHz sampling frequency with 24-bit precision (using the National Instruments NI USB-4431). The EMG linear envelope (EMG-LE) signal was obtained by applying full-wave rectification followed by a low-pass filter (Butterworth 3-rd order, 5 Hz cut-off frequency). The MMG signal was extracted from the FSR sensor’s raw signal by applying a high-pass filter (Butterworth 3-rd order, 2 Hz cut-off frequency). Simultaneous signals were recorded when the subjects performed some voluntary muscle contractions of different intensity and duration.

Some tests were carried out to assess the ability of the FSR sensor to implement the proportional control of a prosthesis. A 3D-printed, five-finger, under-actuated prosthetic hand was used, which was powered with a single motor [43,44,45], and previously controlled by an EMG-LE signal. The control was proportional—the more intense the muscular contraction, the more the hand clenches (performing a synergic grasp movement). The test consisted of replacing the EMG-LE control signal with the raw signal generated by the FSR sensor. Healthy volunteer subjects wore the prosthetic hand and performed some predefined tasks—firstly by using the EMG control and after using the FSR sensor control. Both control signals were acquired from the flexor carpi ulnaris muscle. The predefined tasks included grabbing both non-deformable objects (such as fruits, glasses, screwdrivers, etc.) and deformable objects (such as t-angled cables, sponges, rubber balls, etc.), pouring some water from a plastic bottle into a glass to drink, and catching a flying ball thrown by another person.

## 3. Results

### 3.1. Static and Dynamic Test Results

**Figure 5:**
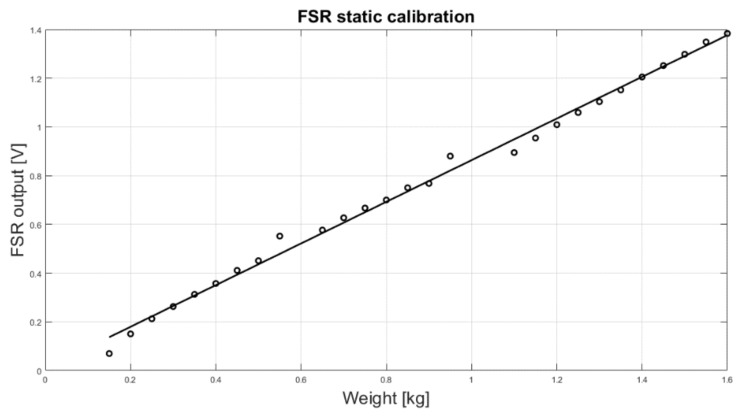
FSR static calibration: scatter plot of the experimental data (o) and regression line.

The results of the static calibration of the FSR are presented in Figure 5. The gain resistor R_G_ (see Figure 2) was set to 700 Ω and the voltage V_FSR_ across the FSR to 2 V. The experimental measurements are represented as circles while the linear regression is represented as a continuous line. The angular coefficient of the regression line was 0.855, whereas the coefficient of determination R^2^ of the linear regression was 0.99, proving a good fitting.

**Figure 6:**
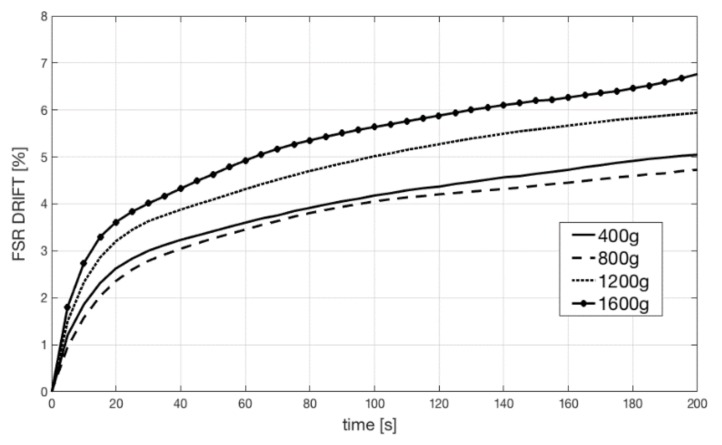
FSR drifts at different constant loads (400, 800, 1200, and 1600 grams).

Figure 6 shows the FSR sensor output drift at different loads. Constant weights of 400, 800, 1200, and 1600 grams were applied onto the sensor for 200 s. The sensor drifts were plotted as a percentage of the expected value. The drift amount did not correlate with loads, but was always confined below 8%. These measurements are compatible with the mechanical model of the sensor [31].

**Figure 7:**
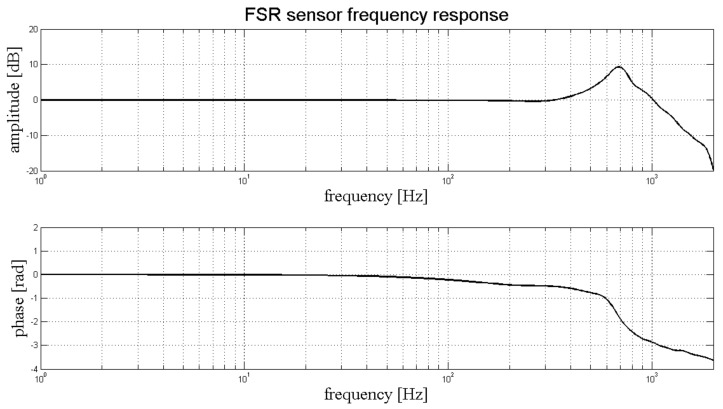
FSR dynamic response: amplitude (upper panel) and phase (lower panel) frequency response.

Figure 7 shows the frequency response of the FSR sensor. We reported the amplitude response in the upper panel, and the phase response in the lower panel. The amplitude response was flat, and the phase linear, up to 300 Hz. A mechanical resonance peak can be observed at about 700 Hz. The bandwidth of the sensor is more than enough to correctly represent the MMG signal, since the human MMG spectrum goes from 2 Hz up to 120 Hz [7,8].

### 3.2. EMG–FSR Comparison Results

**Figure 8:**
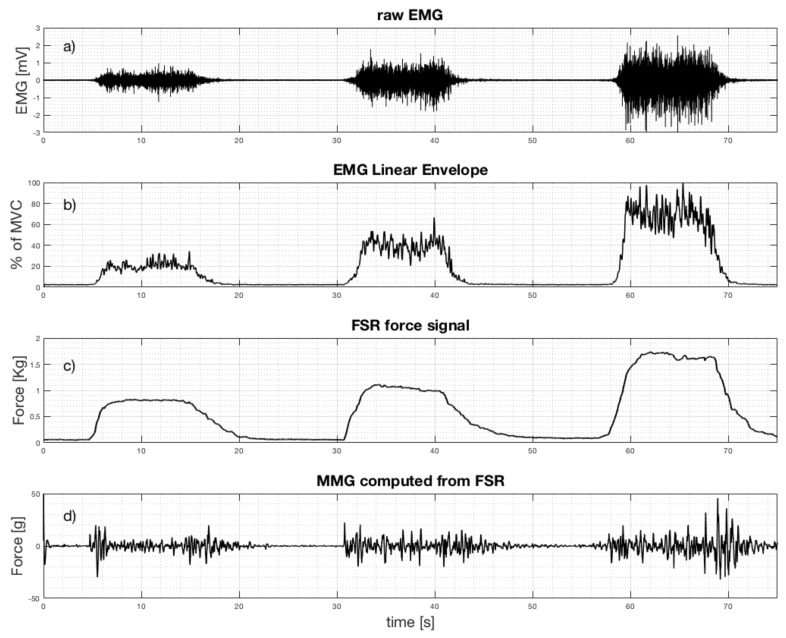
Simultaneous recordings from flexor carpi ulnaris when performing three grasp actions at increasing strength: (**a**) Raw EMG signal; (**b**) EMG linear envelope; (**c**) FSR force signal (raw output); (**d**) mechanomyogram (MMG) extracted from FSR.

The following is some of the results obtained when the healthy subjects performed freehand grasping movements of different intensity and duration. As an example, Figure 8 shows the simultaneous recordings of the EMG and the FSR sensor output, as well as the computed EMG linear envelope and the MMG signal (computed from FSR raw signal). The EMG linear envelope was plotted as a percentage of the maximum voluntary contraction (MVC), while the FSR signals were expressed in kilograms. Three separate muscle contractions of different intensity are clearly evident (each contraction starts at about 5, 31, and 58 s, see Figure 8). The third contraction corresponds to the maximum voluntary contraction (MVC).

There is clearly a good match between the EMG linear envelope and the FSR force signal. The Pearson’s correlation coefficient “r” was computed between these two signals to quantitatively measure their similarity: it scored 0.9286 (*p*-value < 0.0001 (two-tailed test)). However, a delay of the force signal with respect to the EMG is particularly noticeable at the end of each contraction. This is probably due to the electromechanical delay, which is longer during muscle relaxation [34,37]. Thirty contractions of the subjects’ flexor carpi ulnaris were analyzed, and the Pearson’s correlation coefficients computed between the FSR output and the correspondent EMG-LE were always greater than 0.9.

As expected [7,8], the amplitude of the MMG signal corresponding to the central part of muscle contraction (excluding the start and the end) increases with the muscular strength. The standard deviations of the MMG signal computed in the central part of the contractions (i.e., from 7 to 14 s for the first contraction; from 33 to 40 s for the second contraction; and from 61 to 68 s for the third contraction, see Figure 8) resulted in 2.7, 5.2, and 7.0 grams, respectively. The main part of the MMG spectrum results were concentrated at between 2 and 20 Hz, in accordance to literature [7,8].

The FSR force signal was used, instead of the EMG-LE, to implement a proportional control strategy for a prosthetic hand [43,44,45]. Five healthy subjects (not amputees) wore the prosthesis, and after only a few minutes of training, they were asked to perform the predefined tasks. All the subjects were able to successfully perform the assigned tasks, and all subjects reported no appreciable differences between the EMG-LE control and the FSR control of the prosthesis. All subjects also reported that the FSR sensor was easier to wear and less obstructive than the gelled electrodes used for the EMG. In conclusion, these preliminary tests suggest that the new FSR sensor can be a viable alternative to the EMG for controlling the hand prosthesis.

## 4. Discussion and Conclusions

A new, FSR-based sensor able to detect muscle contraction and MMG has been presented in this study. The specific mechanical coupling and conditioning circuit allowed a quantitative and more reliable evaluation of the muscle contraction level with respect to previous studies involving mere FSR sensors [29,30]. The new sensor allows detection of muscle contraction and provides signals that can be compared to EMG–LE (taking into account the electromechanical delay). The new sensor is simple and ready for immediate use, is proportional to muscle contraction level, non-invasive, non-obstructive, easy to wear, robust, unaffected by electromagnetic interferences, and low-cost. Furthermore, it does not make use of electrodes, does not require any electrical contact with patients’ skin, and does not need any signal processing to detect muscle contraction levels (only for MMG computing a simple high-pass filter is required). The FSR-based sensor is much simpler with respect to others (e.g., [19,20,21,22,23,24,25,26,27]) in measuring mechanical muscle variations during contraction.

However, the enclosed FSR shows drift errors caused by the mechanical creep of the polymeric matrix; these drawbacks can be reduced by using an appropriate conditioning circuit for the FSR. No experiment was carried out to evaluate FSR sensor performance during muscle fatigue.

The new sensor was successfully used to implement a proportional control strategy for an underactuated hand prosthesis by providing a valuable replacement for the EMG signal.

In conclusion, the new FSR sensor can effectively monitor muscle contraction intensity and can be used as a valid substitute for EMG-LE to proportionally control prostheses or, more generally, for Human-Machine Interface (HMI) applications. The MMG signal provided by the sensor can be used to control prostheses too [10,12,14,46].

In the future, more research is needed to study the possibility of using even smaller sensors and assembling them into arrays or matrices to model and verify the mechanical cross-talk with surrounding muscles, as well as to study the combined application of EMG and mechanical sensing for diagnosis [39,40,41,47], etc. The simplicity of the sensor and its conditioning circuit allows for easy integration with personal devices for pervasive patient monitoring [4,48]. The FSR sensor could also be used for monitoring muscle performance during whole body vibration treatments [49], and may be suitable for clinical use in applications that do not specifically require EMG recording, such as gait analysis, sport medicine, or others [50].
